# Preliminary Evidence of Apathetic-Like Behavior in Aged Vesicular Monoamine Transporter 2 Deficient Mice

**DOI:** 10.3389/fnhum.2016.00587

**Published:** 2016-11-18

**Authors:** Aron Baumann, Carlos G. Moreira, Marta M. Morawska, Sophie Masneuf, Christian R. Baumann, Daniela Noain

**Affiliations:** Department of Neurology, University Hospital of ZurichZurich, Switzerland

**Keywords:** apathy, depression, PD mice, vesicular monoamine transporter 2, goal-directed behaviors

## Abstract

Apathy is considered to be a core feature of Parkinson’s disease (PD) and has been associated with a variety of states and symptoms of the disease, such as increased severity of motor symptoms, impaired cognition, executive dysfunction and dementia. Apart from the high prevalence of apathy in PD, which is estimated to be about 40%, the underlying pathophysiology remains poorly understood and current treatment approaches are unspecific and proved to be only partially effective. In animal models, apathy has been sub-optimally modeled, mostly by means of pharmacological and stress-induced methods, whereby concomitant depressive-like symptoms could not be ruled out. In the context of PD only a few studies on toxin-based models (i.e., 6-hydroxydopamine (6-OHDA) or 1-methyl-4-phenyl-1,2,3,6-tetrahydropyridine (MPTP)) claimed to have determined apathetic symptoms in animals. The assessment of apathetic symptoms in more elaborated and multifaceted genetic animal models of PD could help to understand the pathophysiological development of apathy in PD and eventually advance specific treatments for afflicted patients. Here we report the presence of behavioral signs of apathy in 12 months old mice that express only ~5% of the vesicular monoamine transporter 2 (VMAT2). Apathetic-like behavior in VMAT2 deficient (LO) mice was evidenced by impaired burrowing and nest building skills, and a reduced preference for sweet solution in the saccharin preference test, while the performance in the forced swimming test was normal. Our preliminary results suggest that VMAT2 deficient mice show an apathetic-like phenotype that might be independent of depressive-like symptoms. Therefore VMAT2 LO mice could be a useful tool to study the pathophysiological substrates of apathy and to test novel treatment strategies for apathy in the context of PD.

## Introduction

About 40% of all patients with Parkinson’s disease (PD) are estimated to suffer from apathy (den Brok et al., 2015), a behavioral syndrome that is characterized by loss of or diminished motivation for goal-directed actions, affecting behavior, emotions and cognition (Robert et al., 2009; Pagonabarraga et al., 2015). Apathy in PD is of clinical relevance, because of its association with poorer prognosis and raised caregiver burden (Pedersen et al., 2009b; Schiehser et al., 2013), and because it is often a chronic condition which is difficult to treat (van Reekum et al., 2005; Benito-León et al., 2012; Skorvanek et al., 2013).

Apathy in PD is highly comorbid with depression and can easily be misinterpreted as such (van Reekum et al., 2005). However, the progression of apathy in PD is rather linked to the progression of the typical motor-symptoms than with depression (Zahodne et al., 2012), probably indicating independent roots for both conditions in the context of PD. In this line, apathy might be associated with a hypodopaminergic state in the mesocorticolimbic pathway in PD (Czernecki et al., 2008; Pagonabarraga et al., 2015), although lesions and neuroimaging studies suggest the dysfunction of frontal-subcortical circuits, especially those linking the ventromedial prefrontal cortex to related regions in the basal ganglia, also to be relevant for the development of apathy (van Reekum et al., 2005; Levy and Czernecki, 2006; Chase, 2011; Santangelo et al., 2013; Robert et al., 2014).

Despite the increasing awareness on the importance of apathy as a comorbid condition in PD, specific treatment strategies for patients are lacking. Furthermore, assessment tests in rodents for preclinical study of apathy are poorly developed. Since apathetic-like symptoms in rodents might be mistaken for depressive-like signs, tests to distinctively assess those symptoms would be favorable. We therefore selected three established behavioral tests, the burrowing test, the nest building test and the saccharin preference test, which we hypothesize might measure apathetic-like symptoms in mice. The burrowing behavior probably constitutes a motivation-dependent behavioral need, which is based in the behavior itself and not on its functional consequences (Sherwin et al., 2004). This finding is akin to human apathetic patients, who show drastically reduced self-generated voluntary and purposeful behaviors (Levy and Dubois, 2006). Moreover, it is suggested that burrowing represents a measure of well-being (Jirkof, 2014) and a murine form of the ability to perform goal-directed “activities of daily living”, like taking care of oneself and others (Deacon, 2009; Jirkof, 2014). The same has been suggested for the nest building behavior (Deacon, 2009; Jirkof, 2014). Because dysfunction of motivation is a core feature of apathy (Robert et al., 2009) and decreased well-being and goal-directed activities are also significantly impaired in patients with apathy (Marin, 1991), we speculate that decreased performance in these tests could possibly reflect an apathetic-like phenotype in mice. The preference for sweet solutions has been proposed to measure anhedonia (Malatynska et al., 2012; Overstreet, 2012; Klein et al., 2015), which is not only a feature of depression, but also of apathy (Pagonabarraga et al., 2015) and has been shown to be more closely associated with apathy than with depression in PD (Kaji and Hirata, 2011). To differentiate apathetic-like from depressive-like symptoms we used the forced swimming test, a widely used test to assess signs of depression in rodents (Borsini and Meli, 1988; Bourin et al., 1998; Cryan et al., 2005; Petit-Demouliere et al., 2005).

In this line, selecting tests that measure dopamine-modulated goal-directed and/or reward-related behaviors is key to reliably assess apathy in animals. For instance, it has been shown that nest building behavior in rodents is disrupted by the administration of dopamine receptor antagonists and restored by application of agonists (Giordano et al., 1990; Silva et al., 2001) or gene therapy for dopaminergic restoration in dopamine-deficient mice (Szczypka et al., 2001). Moreover, the impaired nest building performance in 1-methyl-4-phenyl-1,2,3,6-tetrahydropyridine (MPTP)-lesioned C57Bl/6 mice (Sedelis et al., 2000; Hofele et al., 2001), and the consequential improvement after L-DOPA injections (Sager et al., 2010), emphasize dopaminergic involvement in the test. In the same manner, it has been reported that dopaminergic systems are involved in the preference for a sweetened solution (Muscat and Willner, 1989; Brenes and Fornaguera, 2008), therefore allowing to hypothesize that the saccharin preference test might reflect apathetic-like symptoms by impairments in the reward system.

Here we aim at characterizing an established murine model of PD in terms of apathetic- and possible depressive-like phenotypes in order to potentially provide a novel biological tool for the study of apathy in the context of PD. Thus, we used 12 months old vesicular monoamine transporter 2 (VMAT2) deficient mice (VMAT2 LO mice or LO mice), a well- established animal model of PD (Mooslehner et al., 2001; Caudle et al., 2007; Taylor et al., 2009), to test for an apathy-like phenotype by means of the burrowing test, nest building test and saccharin preference test, and for a possible depressive-like phenotype by means of the forced swimming test. Our preliminary results suggest that for 12 months VMAT2 LO mice show a rather apathetic than depressive-like phenotype, therefore becoming a potential tool for the study of neurobiological substrates and specific treatments for apathy in the context of PD.

## Materials and Methods

### Animals

In this study we tested two different mouse genotypes. The first group consisted of VMAT2 deficient mice (LO, *n* = 15) on a C57Bl/6J background (Mooslehner et al., 2001; Caudle et al., 2007; Taylor et al., 2009), originally obtained by embryonic recovery from Jackson laboratories with the kind permission of Prof. Dr. G. Miller (Emory University, Atlanta, GA, USA) and bred in-house (BZL, University Hospital, Zurich, Switzerland). The second group consisted of wild-type control C57Bl/6 mice (WT, *n* = 19) either littermates or obtained from a supplier (Janvier Labs, Le Genest-Saint-Isle, France). All mice were male and their mean age at the start of the experiment was 371 days with a standard deviation of 8 days and a range of 355–380 days. We single-caged the mice in clear-transparent Eurotype II L plastic cages (Indulab AG, Gams, Switzerland), with food and water available *ad libitum* 7 days prior to behavioral testing. The room temperature was maintained constantly at 21–22°C, and on a 12 h light-dark cycle starting at 8.00 or 9.00 a.m., according to season. Both animal housing conditions and experimental manipulations were approved by the Ethics Committee of the Cantonal Veterinary Office of Zurich, Switzerland under license no. ZH 205/2012, and were in accordance with Swiss Animal Protection Ordinance.

### Genotyping and PCR

We permanently marked VMAT2 LO mice with ear punches following a standard mice numbering procedure. We then used the ear biopsies to extract genomic DNA (DNeasy^®^ Blood and Tissue Kit, Qiagen, Germany) and performed the genotyping by polymerase chain reaction (PCR) experiments. Genotypes were further confirmed from DNA extracted from tail samples taken immediately prior to the sacrifice of the animals. Briefly, the PCR protocol was performed with the forward primer Forward_WT (5′-GCGAATATTCCAGTCCTCCA-3′) and the reverse primer Rev_WT (5′-CAGGCAACACCAGAAACAAT-3′) for VMAT2 WT and Rev_KO (5′-GGAAAGTGAGCCACCATGTAG-3′) for VMAT2 LO. Amplification was performed in a 25 μL reaction mixture with 9.5 μL nuclease free water, 1 μL forward primer (10 μM), 0.5 μl of each reverse primer (10 μM), 12.5 μl GoTaq green master mix (Promega, Madison, WI, USA) and 1 μL of DNA using a standard thermocycler (TProfessional; Biometra, Göttingen, Germany). PCR cycle started with 4 min of initial denaturation at 94°C, followed by 25 repeated cycles of 1 min denaturation at 94°C, 1.5 min annealing at 57°C and 1 min of extension at 72°C, and ended with 7 min of final extension at 72°C. PCR products were stained with GelRed (Biotium, Hayard, CA, USA) separated with electrophoresis at 150 V for 1 h in a 3% agarose gel (w/v) in 1 × TAE buffer and visualized under UV light.

### Behavioral Testing

#### Burrowing Test

The burrowing test has been proven to be sensitive to brain lesions, infections, pharmacological agents, pain, models of inflammatory bowel disease, schizophrenia, anxiety, Alzheimer’s Disease and other pathological states (Deacon, 2006b; Jirkof, 2014). Burrowing behavior is thought to constitute a motivation-dependent behavioral need (Sherwin et al., 2004) that might represent a murine form of the ability to perform “activities of daily living” (Deacon, 2009; Jirkof, 2014). The burrowing test measures how much an animal burrows, i.e., how much burrowing material (e.g., food pellets) of a filled container is removed during a defined time window. Mice achieve the removal by kicks and coordinated hind- and forelimb movements. This burrowing behavior is quantified by comparing the amount of burrowing material in the container at the onset of the experiment with the amount left over after the experiment. We adapted the test to the description of previously published records (Deacon, 2006a; Jirkof, 2014). Briefly, we habituated all mice to the burrowing setup prior to the experiment and performed the test in the home cage. We placed two 250 mL plastic bottles (150 mm × 55 mm, l × Ø) that served as burrowing containers and placed them in the left and right corners of the cages’ posterior walls. In each cage, we filled one container (alternatively left or right) with ~140 g of regular food pellets that served as burrowing material. The other container was left empty and served as shelter. We measured the weight of the filled container before the test (at 18:00 h), 2 h later (2 h; 20:00 h) and on the next morning (overnight; 9:00 h). After the 2 h-measurement, the burrowing container was refilled up to the same weight as at test onset. Percentage of pellets burrowed was calculated separately for the 2 h and the overnight measurement. This was done by subtracting the weight of burrowing container after the test from the weight of burrowing container at test start. Absolute values were then transformed in percentages, resulting in the percentage burrowed for the 2 h and the overnight period. Initially, we tested *n* = 19 WT and *n* = 15 LO mice. After inspection of the data, good and poor burrowers of each genotype and for each test period were identified by median split in order to analyze the groups separately. Thus, proficient burrowers were defined as those presenting burrowing rates ≥ the group median: 2 h period 85% for WT and 73% for LO, overnight period 100% for WT and LO; and poor burrowers were the animals below the median of the respective group. Two outliers were identified using the Grubbs’ test and excluded from the analysis: 1 LO mouse from the 2 h poor burrowers population and 1 WT mouse from the 24 h poor burrowers population. Finally, we performed 2-sided, unpaired *t*-tests comparing: (1) *n* = 19 WT and *n* = 15 LO mice in both periods (*whole population*); (2) *n* = 10 WT and *n* = 8 LO mice for the 2 h period, and *n* = 10 WT and *n* = 8 LO mice for the overnight period (*proficient burrowers*); and (3) *n* = 9 WT and *n* = 6 LO mice for the 2 h period, and *n* = 8 WT and *n* = 7 LO mice for the overnight period (*poor burrowers*).

#### Nest Building Test

The nest building test has been used to detect deficits in nest quality in murine models of PD, Alzheimer’s Disease, brain lesions, obsessive-compulsive disorders, autism, pain, prion infection and other diseases (Greene-Schloesser et al., 2011; Deacon, 2012; Jirkof et al., 2013). Similar to apathy (Czernecki et al., 2008; Santangelo et al., 2013; Thobois et al., 2013), nest building behavior has been shown to depend on dopaminergic pathways (Giordano et al., 1990; Szczypka et al., 2001; Sager et al., 2010; Paumier et al., 2013) and is assumed to represent the ability to perform “activities of daily living” (Deacon, 2009; Jirkof, 2014). The quality of mice nests can be quantified with the nest building test (Deacon, 2006b), simply by providing nesting material (e.g., a compressed cotton piece) overnight and rating the nest quality on the next morning. Additionally, the weight of shredded nesting material can be used as a semi-independent measure of nest complexity (Deacon, 2012). Prior to the experiment, we habituated the mice to the nesting material and performed the nest building test in the home cage, based on previous reports (Deacon, 2006b; Jirkof, 2014). Briefly, we introduced a cotton Nestlet (5 cm × 5 cm, Indulab AG, Gams, Switzerland) in the center of the cage’s frontal wall. In the following morning, we weighed the amount of untorn Nestlet, calculated the difference in weight to the Nestlet at test onset and expressed the value of material shredded in percentage. Also in the next morning, we scored the nest quality on a 5-point scale; 1: Nestlet > 90% intact (Nestlet not noticeably touched); 2: Nestlet 50–90% intact (Nestlet partially torn up); 3: Nestlet < 50% intact (Nestlet mostly shredded, but no identifiable nest site); 4: Nestlet < 10% intact (identifiable, but flat nest) and 5: Nestlet < 10% intact (a “near” perfect nest). Where criteria for the amount of shredded Nestlet and the nest quality did not agree (e.g., a “near” perfect nest with a Nestlet > 50% intact), the difference was split (Deacon, 2012). This way the nest scorings can also have half values. From the *n* = 19 WT and *n* = 15 LO mice tested, we identified active and inactive nesters and separated them by median-split. Thus, animals displaying naturally poor or inactive nesting activity (nesters below the median of the groups for both parameters assessed, i.e., nest score: 4 points for WT and 3 points for LO mice; percentage of Nestlet torn: 91% for WT and 51% for LO mice) were separated from active or good nesters. Finally, we performed 2-sided, unpaired *t*-tests comparing: (1) *n* = 19 WT and *n* = 15 LO mice for both parameters (*whole population*); (2) *n* = 10 WT and *n* = 10 LO mice for the nest score and *n* = 9 WT and *n* = 8 LO for the percentage of Nestlet torn (*active nesters*); and (3) *n* = 9 WT and *n* = 5 LO mice for the nest score and *n* = 10 WT and *n* = 7 LO for the percentage of Nestlet torn (*inactive nesters*).

#### Saccharin Preference Test

The saccharin preference test has been proposed to measure symptoms of anxiety, depression and anhedonia (lack of pleasure; Forbes et al., 1996; Brenes and Fornaguera, 2008; Malatynska et al., 2012; Klein et al., 2015). However, it is highly likely that the saccharin preference test rather measures apathetic-like symptoms. It has been shown that the preference for sweetened solutions in rodents drastically depends on the dopaminergic system (Muscat and Willner, 1989; Szczypka et al., 2001; Brenes and Fornaguera, 2008; Malatynska et al., 2012), which is also a key player in the pathology of apathy (Pedersen et al., 2009a,b; Zahodne et al., 2012). The preference for sweet solutions can be measured by means of the two-bottle preference test (Bachmanov et al., 2001; Sclafani, 2006), by providing one bottle with sweetened solution, and one bottle filled with water. After a defined time window, the intake of each solution is calculated and the preference for the sweet solution is expressed as a ratio in favor of the sweet solution. In the present study we tested the preference for saccharin, also a sweet compound, and based the protocol on previous reports (Bachmanov et al., 1996; Sclafani, 2006). First, we habituated the mice with the test cages, bottles and their content for 48 h prior to the experiment (Sclafani, 2006). The test took place in clear-transparent Eurotype III test cages (425 mm × 266 mm × 155 mm, l × w × h; Indulab AG, Gams, Switzerland). The cages were equipped with sawdust, regular nest building tissue, a bottle of water, a bottle of freshly prepared 0.1% w/w saccharin solution (Sigma-Aldrich Chemie GmbH, Buchs, Switzerland) and regular food *ad libitum*. Both tap water and saccharin solution were provided in 100 mL plastic drinking bottles with an 8 mm diameter, 2 mm opening and 6.5 cm length stainless steel sipper spout (Techniplast S.p.A., Buguggiate, Italy). We introduced the previously weighed bottles into the test cages and weighed them again 24 h later. We calculated the absolute liquid intake separately for water and saccharin solution by subtracting the weight of the bottles after the test from the weights of the bottles prior to the test. For further analyses, we transformed the weight values into the corresponding volumes and normalized the volumes by the body weight of the respective mice. The saccharin preference ratio was calculated by dividing the volume of saccharin solution drunk by the volume of total liquid consumption. Out of *n* = 19 WT and *n* = 15 LO mice tested, one data point was excluded from the analysis due to leakage of one of the testing bottles. We analyzed the data by a 2-sided, unpaired *t-test* for each variable, incorporating totally *n* = 19 WT and *n* = 14 LO animals.

#### Forced Swimming Test

The forced swimming test is widely used to assess depressive-like symptoms in rodents (Porsolt et al., 1979). In the forced swimming test, mice are forced to swim in a container filled with water that does not allow for escape. Normally they rapidly stop attempts to escape and become immobile, a behavior that is quantified as the time spent immobile, which is typically interpreted as behavioral despair and a measure of depressive-like behavior in rodents. We slightly adapted the forced swimming test as described previously (Porsolt et al., 1979; Taylor et al., 2009). Briefly, we tested the mice in batches of 3–4 animals at a time and their swimming activity was recorded during 4 min from above by an infrared camera. Raw data was acquired and analyzed with the software EthoVision XT 9.0 (Noldus Information Technology GmbH, Oberreifenberg, Germany) on a standard PC. The experimental setup consisted of a set of four square areas (each 53 cm × 53 cm) separated by wooden walls, allowing testing of four mice simultaneously. Immediately before testing, a 2000 mL glass beaker (19 cm × 13 cm, h × Ø) was placed in each area, and filled with 1500 mL fresh (25°C) tap water. Swimming behavior was recorded for 6 min, but only the last 4 min were subjected to analysis (Taylor et al., 2009). We defined the time spent immobile as a floating behavior with a max speed of 3 cm/s, a value that became apparent of being a threshold value for active swimming behavior by detailed analysis of movement tracking. We tested a total of *n* = 19 WT and *n* = 15 LO mice. After inspection of the data, good and poor swimmers of each genotype were identified by median split in order to analyze the sub-groups separately. Thus, proficient swimmers were defined as those presenting immobility times ≤ the group median: 192 s for WT and 194 s for LO. Finally, we performed 2-sided, unpaired *t*-tests comparing: (1) *n* = 19 WT and *n* = 15 LO mice (*whole population*); (2) *n* = 9 WT and *n* = 7 LO mice (*proficient swimmers*); and (3) *n* = 10 WT and *n* = 8 LO mice (*poor swimmers*).

## Results

### VMAT2 Deficient Mice Present Impaired Performance in Goal-Directed and Reward-Related Behavioral Tasks

**Figure 1:**
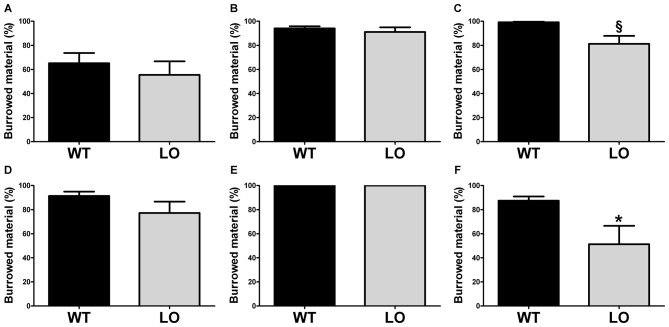
**Decreased burrowing activity in vesicular monoamine transporter 2 (VMAT2) deficient mice.** Burrowing activity was determined 2 h **(A–C)** and one night **(D–F)** after the test start in both LO mice and their WT controls. No differences were found in the whole (*n* = 19 WT, *n* = 15 LO; **A,D**) or proficient (*n* = 10 WT, *n* = 8 LO; **B,E**) burrower populations. In the poor burrower population, the percentage of burrowed material of LO mice was not significantly different from WT mice (*n* = 9 WT, *n* = 6 LO; ^§^*p* = 0.055; **C**) after 2 h. However, the percentage of burrowed material was significantly reduced in LO mice (*n* = 8 WT, *n* = 7 LO; *p < 0.05; **F**) for the overnight period. Bars indicate the mean of the groups and error bars indicate the S.E.M. ^§^Indicates a non-significant trend.

**Figure 2:**
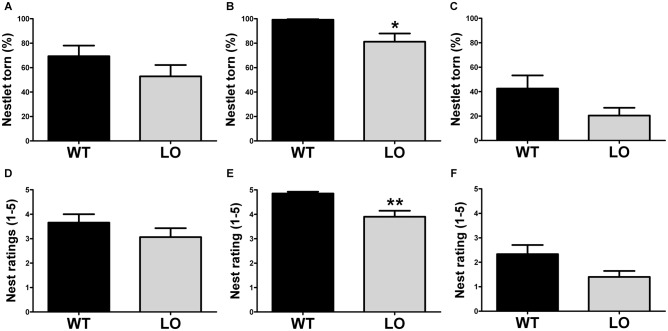
**Impaired nesting in VMAT2 deficient mice.** Nesting performance was assessed by measuring the percentage of the Nestlet torn **(A–C)** and the nest rating (score 1–5; **D–F**) in both LO mice and their WT controls. No differences were found in the whole (*n* = 19 WT, *n* = 15 LO; **A,D**) or inactive (*n* = 9–10 WT, *n* = 5–7 LO; **C,F**) nester populations. However, both the % of Nestlet torn and the nest rating scores were significantly reduced in the LO group within the active nesters population, as compared to their WT controls (percentage of Nestlet torn: *n* = 9 WT, *n* = 8 LO, *p < 0.05, **(B)** nest ratings: *n* = 10 WT, *n* = 10 LO, **p < 0.01, **E**). Bars indicate the mean of the groups and error bars indicate the S.E.M.

To evaluate whether goal-directed behaviors were affected in VMAT2 mutant animals, we tested LO mice and their WT controls in two different tasks, the burrowing test and the nest building test. First, we performed the burrowing test (Figure 1), by which we measured the amount of burrowed material during two different periods of time, 2 h (Figures 1A–C) and overnight (Figures 1D–F) for the whole population tested (Figures 1A,D), the proficient burrowers (Figures 1B,E) and the poor burrowers (Figures 1C,F). Our results showed no significant difference between LO and WT mice within the whole population in the percentage of burrowed food pellets 2 h or 24 h after the test start. In an attempt to reduce the variability and unmask possible differences in subpopulations of animals classified on the basis of their performance, we then identified and separated proficient from poor burrowers in both time periods. In the analysis of the good burrowers no significant differences were observed between genotypes at any test time point. However, in the analysis of the poor burrowers, we observed a non-significant trend towards reduced burrowing performance in LO mice at 2 h (2-sided, unpaired *t*-tests; 2 h WT vs. LO ^§^*p* = 0.055). And we determined a significantly reduced percentage of material burrowed in LO mice, as compared to WT animals (2-sided, unpaired *t* tests; 24 h WT vs. LO *p < 0.05) in the overnight measure. We then performed the nest building test (Figure 2), in which we assessed the nesting activity by measuring the amount of nesting material used (percentage of Nestlet torn, Figures 2A–C) and rated the quality of the nests (rating scores from 1 to 5 points, Figures 2D–F) for the whole population (Figures 2A,D), the active nesters (Figures 2B,E) and the inactive nesters (Figures 2C,F). Our results showed no significant differences for both the whole population and the inactive nesters groups for both tested parameters. However, we observed both significantly lower percentage of Nestlet torn and nest rating in LO mice, as compared to WT controls, in the active nesters group (2-sided, unpaired *t*-tests; percentage of Nestlet torn WT vs. LO *p < 0.05, nest ratings WT vs. LO **p < 0.01).

**Figure 3:**
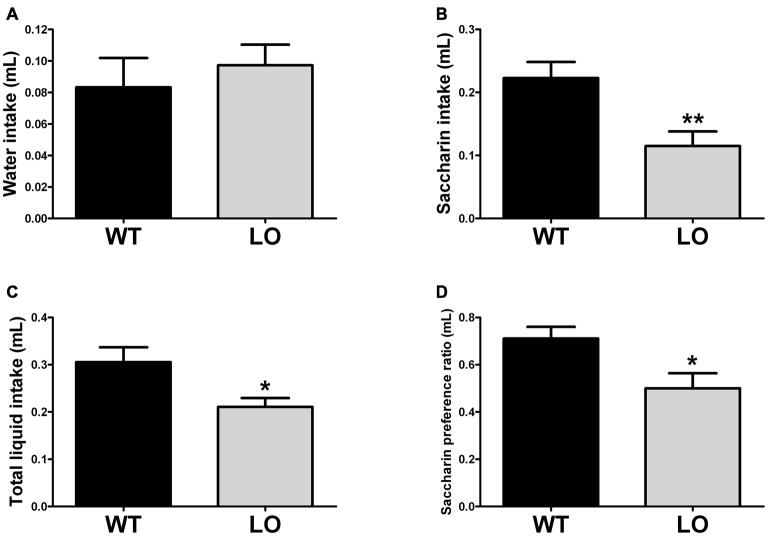
**Reduced preference for a sweetened solution in VMAT2 deficient mice.** The amount of water **(A)** saccharin **(B)** and total liquid **(C)** intake was determined by the saccharin preference test. In addition, we calculated **(D)** the saccharin preference ratio as the intake of (saccharin/(saccharin + water)). There was no difference in water intake between LO and WT mice. However, saccharin and total liquid intake (driven by the reduction in saccharin intake) was significantly reduced in LO mice, as compared to their WT controls (saccharin intake **p < 0.01, total liquid intake *p < 0.05). A significantly reduced saccharin preference ratio was also driven by the pronounced reduction in saccharin consumption (*p* < 0.05). All measures were normalized by the respective body weight to control for the lower weight of the VMAT2 mutants. Bars indicate the mean of the groups and error bars indicate the S.E.M.

To explore the reward-related behavioral performance in VMAT2 mutant animals, we subjected LO and WT mice to the saccharin preference test (Figure 3). In the SPT we measured water (Figure 3A), saccharin (Figure 3B) and total liquid intake (Figure 3C) in respect to body weight, as well as the saccharin preference ratio (Figure 3D) in both genotypes. Our results showed similar water intake of LO and WT mice. However, total liquid intake, saccharin intake and the saccharin preference ratio were significantly reduced in LO animals, as compared to their WT controls (2-sided, unpaired *t*-tests; water intake WT vs. LO *p* > 0.05, saccharin intake WT vs. LO **p < 0.01, total liquid intake WT vs. LO *p < 0.05, saccharin preference ratio WT vs. LO *p < 0.05).

### VMAT2 Deficient Mice Do not Present Evidence of Depressive-Like Behavior

**Figure 4:**
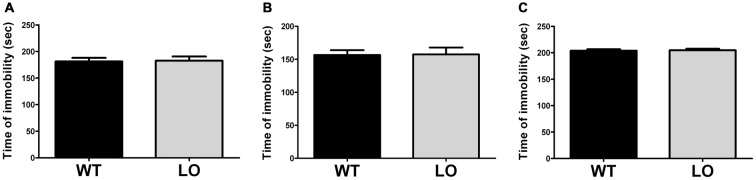
**Normal immobility time in VMAT2 deficient mice.** The time of immobility during a forced swimming session of 4 min in WT and LO mice indicated no significant differences between both genotypes in the whole and the two sub-populations analyzed. Two-sided, *t* test; **(A)** whole population: *p* = 0.896; **(B)** proficient swimmers: *p* = 0.742; **(C)** poor swimmers: *p* = 0.777. Bars indicate the means of the groups and error bars indicate the S.E.M.

To determine the existence of depressive-like behavior in LO mice, we subjected them and their WT controls to the forced swimming test (Figure 4). Using an automated recording system, we measured the time spent immobile of each animal during a forced swimming session of 4 min. Our results showed similar immobility times in LO and WT mice in the whole and the two sub-populations analyzed (2-sided, unpaired *t*-tests; time of immobility WT vs. LO *p* > 0.05).

## Discussion

In this study we aimed at evaluating the usefulness of VMAT2 deficient mice as a tool to study apathy-like symptoms (i.e., reduction in goal-directed, reward-related behaviors) in the context of PD.

Although the pathological mechanisms of apathy are poorly understood and need further elucidation (Santangelo et al., 2013; Pagonabarraga et al., 2015), impairment in the dopaminergic pathways are assumed to underlie both PD and apathy (Pedersen et al., 2009a,b; Zahodne et al., 2012). Thus, animal models mimicking PD pathology are of special interest in the study of apathy. However, since apathy and depression are closely related but yet different constructs, depression can be a confounder in the study of apathy, especially in the context of PD, where depression and apathy are both common comorbid disorders (van Reekum et al., 2005). Thus, animal models of PD presenting apathy-like symptoms in the absence of depressive-like symptoms are desirable for the study of apathy’s underlying processes and the development of specific treatments.

We evaluated the performance of 12 months old VMAT2 LO mice in four behavioral tests, namely the burrowing test, nest building test, saccharin preference test and forced swimming test, and observed a significantly lowered performance in LO mice in the first three behavioral tasks. The reduced burrowing and nest building activities suggest impairment in goal-directed, motivated behaviors in VMAT2 LO mice. Burrowing likely constitutes a motivation-dependent behavioral need (Sherwin et al., 2004) and nesting, as well as burrowing behavior, are suggested to represent a murine form of the ability to perform goal-directed “activities of daily living” (Deacon, 2009; Jirkof, 2014). These impairments might be akin to human apathetic patients, who show drastically reduced self-generated voluntary and purposeful behaviors (Levy and Dubois, 2006), and dysfunction in motivation (Robert et al., 2009). The saccharin preference test might also reflect apathetic symptoms in VMAT2 LO mice by reflecting the reduced preference for sweet solutions as a consequence of impaired mesolimbic dopaminergic reward pathways. Mice treated with dopamine receptor antagonists or injected with 6-hydroxydopamine (6-OHDA) into the nucleus accumbens exhibit significantly reduced preference for sweet solutions (Muscat and Willner, 1989; Malatynska et al., 2012). Vice versa, the restoration of dopamine production in the nucleus accumbens of dopamine-deficient mice could normalize the preference for sweet solutions (Szczypka et al., 2001). Lesions of the ventral tegmental area in mice also entailed diminished sucrose preference (Martínez-Hernández et al., 2006), and the consumption of sucrose in rats has been shown to induce the release of dopamine and to correlate with its concentration in the ventral striatum (Brenes and Fornaguera, 2008; de Araujo et al., 2008). The circuit of dopaminergic projections from neurons of the ventral tegmental area to the ventral striatum, more precisely to the nucleus accumbens, is indeed, the best characterized reward circuit in the rodent brain (Russo and Nestler, 2013). However, dopamine is not the only afflicted neurotransmitter in apathy, neither in PD nor in VMAT2 LO mice. In VMAT2 LO mice norepinephrine concentrations are also drastically reduced, illustrated by progressive degeneration in the locus coeruleus, that even preceded degeneration in the substantia nigra pars compacta (Taylor et al., 2014). A recent study revealed that at the age of 4–6 months VMAT2 LO mice also exhibited dramatically reduced serotonin release capacity (Alter et al., 2015). Thus, reducing the VMAT2 LO model primary to a dopaminergic deficiency is oversimplifying, and the same criticism can be applied to the hypothesis of apathy being mainly caused by dopaminergic dysfunction. Apathy is a complex syndrome that is not limited to dopaminergic activity, as serotonergic, cholinergic and noradrenergic neurotransmitter systems are known to be involved in the same circuits that are affected in PD (Pagonabarraga et al., 2015).

On the other hand, our forced swimming test result showed that 12-months old VMAT2 LO and WT mice do not differ in the time spent immobile during the 4 min of the test, indicating a possible lack of depressive-like symptoms in the VMAT2 mutants. In addition, own results in the tail suspension test indicate a similar finding, with unchanged time spent immobile in the mutants as compared to controls (data not shown). These findings are conflicting with previously reported data from VMAT2 LO and HET mice, showing increased immobility times in the forced swimming test (Fukui et al., 2007; Taylor et al., 2009). Specifically, VMAT2 HET mice showed increased immobility times at the age of 3–5 months (Fukui et al., 2007), whereas the VMAT2 LO mice did not show increased immobility times at 4–6 months, but only later at the age of 12–15 months (Taylor et al., 2009). Despite these conflicting data, a series of aspects confirm the validity of our findings. First, the previously characterized VMAT2 HET mice differ in age and genotype from the LO mice tested here and, therefore, our results cannot be directly contrasted to those of Fukui et al. (2007). Second and in regard to the observations of Taylor et al. (2009), it can be speculated that depressive-like symptoms could start evidencing only after 12 months of age and therefore manifest later on in increased immobility times. Finally, our sample sizes were significantly larger than in the previous VMAT2 LO characterization, providing increased validity to our results.

Dysfunctions of the nigrostriatal, mesolimbic and mesocortical dopaminergic pathways are suggested to play an important role in the manifestation of apathy (Thobois et al., 2010; Santangelo et al., 2013). Indeed, several findings substantiate the importance of dopaminergic activity in the pathology of apathy. Dopaminergic treatment seems to decrease apathy in early stages of PD (Santangelo et al., 2014) and the severity of apathy changes in regard to the dopaminergic “on” and “off” treatment states (Lhommée et al., 2012; Castrioto et al., 2014). Since VMAT2 deficient mice are vastly impaired in their dopaminergic neurotransmission, it is conceivable that they would manifest symptoms of apathy.

The main limitation of our study is the missing longitudinal dimension in our experiments. Evaluating the progression of apathetic symptoms throughout the life-span of the mice, as well as its temporal relation with depressive-like symptoms would provide a more profound insight regarding the temporal window of apathy-based studies and the development of specific treatment options for each condition. An additional drawback of our design is that the applied behavioral tests may not be entirely specific for one symptom or another. In this line, the independence of apathy from depression has been matter of intense debate, as apathy has classically been considered as a core feature of depression (Kirsch-Darrow et al., 2006; Santangelo et al., 2013) and many definitions of depression comprise features of apathy (Cummings et al., 2015). Moreover, we explore apathy in an animal model of dopaminergic deficiency rather than implementing a specific test for apathy, which remains largely missing in the field. Thus, relying on behavioral tests that accurately measure the effect of dopaminergic dysfunction over goal-directed and reward-related behaviors in the context of PD, appears as a risky but promising strategy in the study of pure apathy.

In summary, 12 months old VMAT2 deficient mice likely do not express a depressive-like phenotype but rather show behavioral signs of apathy that might be related to dopaminergic dysfunction. Future research should validate our preliminary results and address potential modulatory effects of dopaminergic treatment on the performance in the burrowing, nest building and saccharin preference tests, and closely relate the development of the apathetic phenotype to the underlying neurochemical changes in VMAT2 deficient mice’s brains.

## Author Contributions

DN and CRB designed research. AB, CGM, MMM and SM executed research. AB and DN analyzed data, prepared the figures and wrote the manuscript. All authors edited the manuscript.

## Funding

The study was funded by the Clinical Research Priority Program “Sleep and Health” of the University of Zurich (UZH) and by the HSM-2 Initiative “Stereotactic Neurosurgery” of the Canton of Zurich.

## Conflict of Interest Statement

The authors declare that the research was conducted in the absence of any commercial or financial relationships that could be construed as a potential conflict of interest.
